# Assessing the consistency between the anthropometric method and bioelectrical impedance analysis when calculating the Heath-Carter somatotype in people without obesity: a cross-sectional study

**DOI:** 10.2478/joeb-2025-0013

**Published:** 2025-08-04

**Authors:** Kyialbek Sakibaev, Alexander Meshtel, Tatyana Grichanova, Kylychbek Suiunov, Abdyrakhman Eshiev, Sagynbek Dzholdubaev, Nazgul Tashmatova, Mirlan Nuruev, Aiperi Alimbekova, Akmaral Argynbaeva, Uulkan Manas Kyzy, Bekbolot Keneshbaev, Kulpunai Karimova

**Affiliations:** Department of Natural Sciences and Humanities, Osh International Medical University, Osh, Kyrgyzstan; Department of Anatomy and Biological Anthropology, Russian University of Sports, Moscow, Russian Federation; Department of Normal and Topographical Anatomy, Medical Faculty, Osh State University, Osh, Kyrgyzstan; Department of Anatomy, Histology and Normal Physiology, International Medical Faculty, Osh State University, Osh, Kyrgyzstan; CARDIO ASIA-PLUS Medical Center, Osh, Kyrgyzstan; Scientific Research Institute of Biomedical Problems of the Southern Branch of the National Academy of Sciences, Osh, Kyrgyzstan

**Keywords:** Anthropometry, bioelectrical impedance analysis, somatotyping, anatomy, Heath-Carter somatotype, consistency

## Abstract

Body physique assessment is important for various fields of science and practice, including anthropology, medicine, sports nutrition and pedagogy. The classic Heath-Carter method is complex and multi-step, while bioelectrical impedance analysis makes it easier and faster to evaluate this parameter. The purpose of this study is a comparative analysis of the anthropometric method and bio-electrical impedance analysis in assessing the somatotype. Differences and a low degree of agreement were found between these methods in determining the somatotype.

**Study design:**

Studies were carried out at the Department of Anatomy and Biological Anthropology of the Russian University of Sports “SCOLIPE” (Russia) and the morphological departments of the medical faculties of Osh State University (OshSU) of the Kyrgyz Republic from February to April 2024.

**Results:**

Bioelectrical impedance analysis quickly and more significantly speeds up the process of assessing a person's body type.

**Conclusion:**

Bioimpe-dance analysis shows an increased level of mesomorphy scores in both males and females, and the difference in males is more pronounced. On the other hand, endomorphy scores were lower when assessing the somatotype using the anthropometric method.

## Introduction

Personalized medicine, based on an individual approach to patients, is one of the priorities in the development of modern medical science. The basic method for assessing physical development, nutritional status, and determining the level of health with this approach should be the method of constitutional analysis, i.e., somatotyping, which allows when analyzing a population, to identify whether an individual belongs to different somatotypes [1]. It should be noted that the assessment of physique is an integral part of the intersection of such sciences as anthropology and medicine. At the same time, the anthropometric method for determining the somatotype, repeatedly tested and standardized, is capable of providing objective digital materials when examining an object and does not require significant time and economic costs [2]. However, the classical method of calculating the somatotype according to Heath-Carter [3,4,5,6] has some limitations, which consist of the complexity of measurements and the multi-level process of calculating somatotype scores. Today, traditional anatomical and anthropometric approaches are complemented by effective high-tech research methods, expanding the possibilities of objective assessment of the patient's physical and nutritional status [7].

Bioelectrical impedance analysis (BIA) has found application in epidemiological studies of population health in the European Region of WHO and the European Union (MONICA, NUGENOB), China (KSCDC), USA (Framingham Heart Study, NHANES), South Korea (KNHANES) and other countries, and is one from the methods routinely used in Russian institutions such as health centers. Based on the analysis and processing of primary data from bioimpedance measurements in health centers, centile curves of age-sex variability of anthropometric characteristics and body composition parameters of the Russian population were constructed and estimates of the prevalence of nutritional status disorders and morbidity risks were obtained.

BIA, being a simpler, faster and more accessible method, significantly speeds up the process of assessing a person's body type. In his 2022 work, M.M. Semenov and co-authors conducted a comparative analysis, during which they assessed the differences between the anthropometric method and the BIA method in assessing the somatotype in obese patients [8], while E.V. Chaplyginova et al. [9] compared methods for assessing the somatotype of children. However, we did not find any concordance between these methods for people with normal fat mass.

Consequently, standardization of methods for determining body type and assessing body composition is relevant, since the results of measuring body composition using classical anthropometry in comparison with the hardware method of bioimpedance analysis often differ, and it is important for practicing researchers in the field of sports medicine and nutrition, anthropology and pedagogy to have information about these differences, which was the purpose of this study.

Hence, the purpose of the study is to conduct a comparative analysis of the results of assessing the somatotype according to Heath-Carter using the anthropometry methods and bioelectrical impedance analysis.

## Materials and methods

The studies were carried out at the Department of Anatomy and Biological Anthropology of the Russian University of Sports “SCOLIPE” (Russia) and the morphological departments of the medical faculties of Osh State University (OshSU) of the Kyrgyz Republic from February to April 2024. All subjects provided written informed consent, which stated the purpose and possible risks and information that one could stop participating in the study at any time. The study was approved by the Ethics Committee of Russian University of Sport (No2612, 19.04.2024), Osh State University (No14, 29.06.2024).

The participants of the study were first-year students of the Russian University of Sports “SCOLIPE” and medical faculties of Osh State University, representatives of the Department of Theory and Methodology of Basketball, as well as the Department of Theory and Methodology of Fencing, Modern Pentathlon and Shooting Sports. The study included 25 females and 20 males aged 17–21 years. The sample was formed without strict planning, based on practical possibilities.

Exclusion criteria: taking any medications or dietary supplements that are incompatible with the study and eating the last two hours before the measurement. The females were measured on days 9–11 of the ovarian-menstrual cycle, body fat mass is above 20% in men and 28% in women, according to the World Health Organization (WHO).

All measurements were carried out in the same chronology:

1) Measurement of body weight and anthropometric data.
2) BIA.

Anthropometric measurements were carried out according to the guidelines of the International Society for the Advancement of Kinanthropometry (ISAK) [10]. The skinfold measurement was performed using a Lange caliper. The measurement was carried out three times and the folds of the triceps, medial-calf and iliac crest were used. The mean value of the three measurements was taken as the final value.

**Fig. 1:**
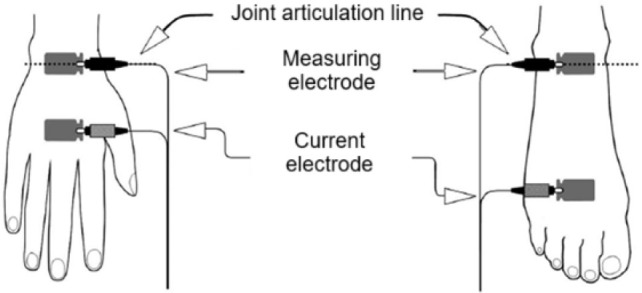
Diagram of the location of measuring (potential) and current electrodes on the patient's hand and foot.

The analysis was performed using the Medass ABC-01 device (Russia), using the hand-foot electrode application method. The measurements were carried out in a supine position. Standardization of the measurement included nutrition control (eating no earlier than two hours before the measurement), exercise control (physical activity no earlier than 24 hours before the measurement) and control of procedures affecting the body's hydration level (going to baths and saunas, massages and other procedures no earlier than 48 hours before the measurement). To confirm compliance with the standard measurement conditions, participants were interviewed before the measurement about violations of the above requirements. During the measurement, the electrodes (Fiab 22 × 34 mm, Italy) were located on the back of the wrist and palm (2 electrodes), as well as on the ankle joint and the back of the foot (2 electrodes), as indicated in the manufacturer's recommendation [11] (Fig. 1). The measurement time was 15–25 seconds.

The calculation of the somatotype using BIA was carried out by software (NTC Medass LLC, Russia) developed by V.A. Kolesnikov and co-authors [12]:

Endomorphy = −2875/R50 + 0.625 × BMI − 0.042 × BM − 0.23 × Sex − 2.33
Mesomorphy = 1467/R50 + 0.552 × BMI − 0.096 × BM + 0.59 × Sex − 4.22
R50 – active resistance (Ohms) at a frequency of 50 kHz,
BMI – body mass index (kg/m^2^),
BM – body mass (kg),
Sex: 1 (male), 0 (female).

Ectomorphy were assessed using weight and height indices.

Statistical data processing was carried out using the Statistica 10 package (StatSoft, USA). The Shapiro-Wilk test was used to assess the normality of distribution. A comparative analysis between methods for assessing somatotype was carried out using Student's t-criterion for dependent samples.

Lin's correlation coefficient of concordance (pc) with 95% confidence interval (CI) and Pearson's correlation coefficient (r) were used to identify the relationship and consistency between the results of somatotype assessment using the BIA and the anthropometric method.

The level of consistency was assessed as almost perfect consistency (pc > 0.99), good consistency (pc = 0.95 – 0.99), weak consistency (pc = 0.90 – 0.94) or no consistency (pc < 0.90). The level of connection for r was assessed using the Chaddok scale: correlation was considered strong when r > 0.9, significant when r = 0.7 – 0.9, noticeable when r = 0.5 – 0.7, moderate when r = 0.3 – 0.5 and weak at r < 0.3.

Measurement bias was assessed using the Bland-Altman method. The p < 0.05 level was found to be statistically significant for statistical tests.

### Informed consent

Informed consent has been obtained from all individuals included in this study.

### Ethical approval

The research related to human use has been complied with all relevant national regulations, institutional policies and in accordance with the tenets of the Helsinki Declaration and was approved by the Ethics Committees Osh State University and RUS «GTSOLIFK».

## Results

Participant data are presented in Table 1.

**Table 1:** Study participant data.

**Parameters**	**M** M ± SD	**F** M ± SD
Height, cm	182 ± 12	164 ± 6
Body mass, kg	61.4 ± 11.7	78.9 ± 15.5
BMI, kg/m^2^	22.3 ± 2.8	24.2 ± 3.9
Age, years	18.1 ± 1.3	18.4 ± 0.9
Shoulder girth, cm	27.3 ± 3.26	34.6 ± 3.7
Calf circumference, cm	34.8 ± 2.3	36.6 ± 3.3
Hip circumference, cm	69.7 ± 8.5	78.6 ± 7.9
Diameter of the distal epiphysis of the humerus, cm	5.3 ± 0.5	6.7 ± 0.6
Diameter of the distal femoral epiphysis, cm	8.1 ± 1.0	9.4 ± 0.8
Subscapular, mm	16.3 ± 7.0	14.3 ± 8.8
Triceps, mm	16.0 ± 5.6	9.9 ± 4.6
Iliac crest, mm	16.1 ± 8.0	13.8 ± 10.3
Medial calf, mm	19.5 ± 8.3	11.9 ± 5.4

***Notes:*** M – Male, F – Female, *Subscapular* – fold of skin and fat under the lower angle of the scapula in an oblique direction at an angle of 45° to the line of the spine, *Triceps* – vertical fold of skin and fat, on the back of the shoulder, in the middle between the olecranon and acromion process, on a freely lowered arm, *Iliac crest* – a diagonal fold passing directly above the iliac crest in a vertical line from the mid-axillary line, *Medial calf* – vertical skinfold, on the medial side of the leg at the level of the maximum circumference.

The data obtained during the comparative analysis are presented in Table 2.

**Table 2:** Results of a comparative analysis of somatotype assessment using anthropometric measurements and bioelectrical impedance analysis.

Female					

**Somatotype**	**BIA**	**Anthropometry**	**p**	**p_c_**	**r**
	M ± SD	M ± SD			

Endo	4.2±1.1	4.6±1,3	0.028^*^	0.84 (0.62; 0.94)	0.89
Meso	4.9±1.0	4.1±0.3	0.001^*^	0.31 (0.12; 0.48)	0.81
Ekto	2.3±1.2	2.3±1.4	0.886	0.96 (0.90; 0.99)	0.97

Male					

**Somatotype**	**BIA**	**Anthropometry**	**p**	**p_c_**	**r**
	M ± SD	M ± SD			

Endo	2.1±1.3	3.1±1.1	0.432	0.21 (−0.40; 0.69)	0.23
Meso	5.6±1.6	3.8±1.4	0.001^*^	0.42 (0.10; 0.66)	0.84
Ecto	2.4±1.3	2.4±1.2	0.167	0.98 (0.94; 0.99)	0.99

***Notes:*** BIA – bioelectrical impedance analysis, pc – Lin's concordance correlation coefficient, r – Pearson's correlation coefficient, ENDO – endomorphy scores, MESO – mesomorphy scores, ECTO – ectomorphy scores,

* – statistically significant differences between measurement methods at p < 0.05.

**Fig. 2:**
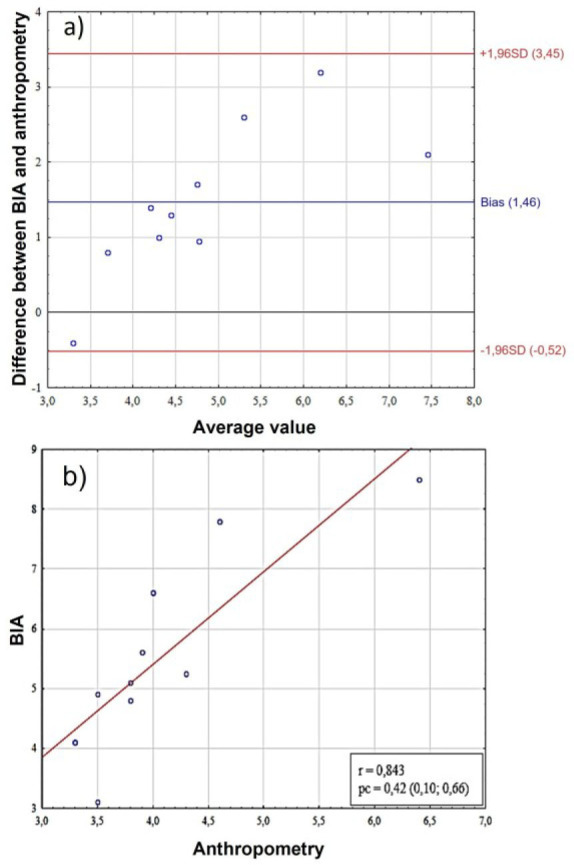
Comparative analysis of endomorphy scores in female. A – assessment of bias using the Bland-Altman method, the Y-axis shows the “BIA-Anthro” difference in endomorphy scores, B – scatter plot between two methods for assessing somatotype. BIA – bioelectrical impedance analysis, SD (standard deviation), r – Pearson correlation coefficient, pc – Lin correlation concordance coefficient.

**Fig. 3:**
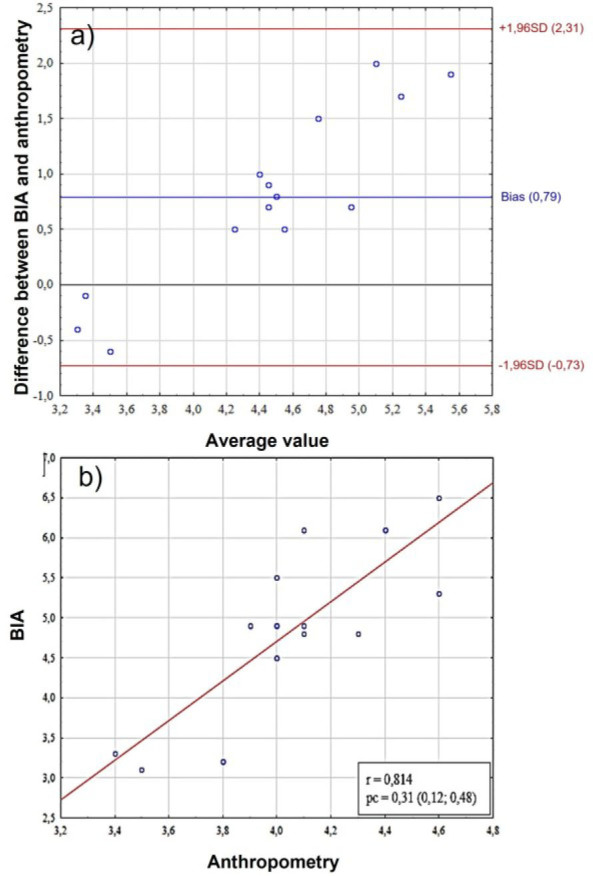
Comparative analysis of mesomorphy scores in females. A– assessment of bias using the Bland-Altman method, the Y-axis shows the “BIA-Anthro” difference in mesomorphy scores, B – scatter plot between two methods for assessing somatotype. BIA – bioelectrical impedance analysis, SD (standard deviation), r – Pearson correlation coefficient, pc – Lin correlation concordance coefficient.

**Fig. 4:**
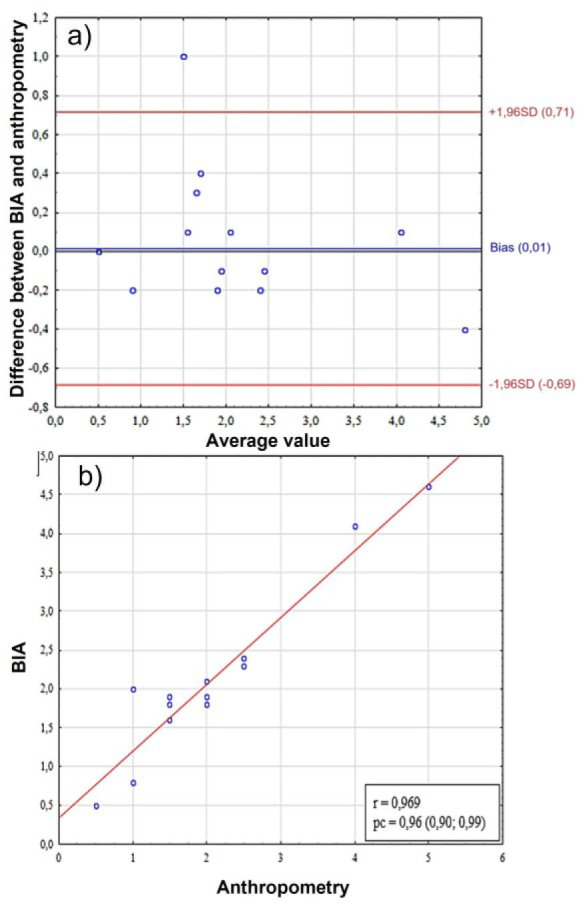
Comparative analysis of ectomorphy scores in females. A – assessment of bias using the Bland-Altman method, the Y-axis shows the “BIA-Anthro” difference in ectomorphy scores, B – scatter plot between two methods for assessing somatotype. BIA – bioelectrical impedance analysis, SD (standard deviation), r – Pearson correlation coefficient, pc – Lin correlation concordance coefficient.

As a result of assessing the somatotype of females based on mesomorphy and endomorphy, no consistency was found between anthropometric methods and BIA. When assessing endomorphy scores, the bias between measurement methods was −0.37 (95%CI = −1.59; 0.78), while for mesomorphy it was 0.80 (95%CI = −0.73; 2.31). The lack of concordance is indicated by a low level of pc (Table 2), and as a result of a comparative analysis, it was revealed that statistically significant differences are present when comparing endomorphy and mesomorphy scores measured in two ways (p < 0.01). The results of both methods showed moderate and good correlations for female's ecto- meso- and endomorphy (table 1, Fig. 2B, Fig. 3B, Fig. 4B)

The analysis of the Bland-Altman graph (fig. 2A) demonstrates the presence of a systematic bias (bias = 1.46) between the methods of BIA and anthropometry, with the limits of agreement ranging from −0.52 to +3.45, indicating a significant variation in differences between the methods. The high correlation coefficient (r = 0.843) between the difference of the methods and their average values indicates a pronounced proportional error, that is, the differences between the methods increase with the increase in the measured value. This is also confirmed by the concordance coefficient (pc = 0.42), which, despite its moderate value, in combination with a high correlation emphasizes the dependence of discrepancies on the level of the measured parameter (Fig. 2B).

The Bland-Altman graph (Fig. 3A) shows a systematic bias between the methods (bias = 0.79) with agreement limits from −0.73 to +2.31. A high correlation (r = 0.814) between the method difference and the average values indicates a proportional error – the discrepancies between the methods increase with increasing measured parameter. The concordance coefficient (pc = 0.31) confirms the moderate consistency of the methods, but with a clear dependence of the differences on the magnitude of the measurements (Fig. 3B).

An analysis of the ectomorphy score results revealed a high level of concordance (pc = 0,96, 95%CI = –0,69; 0,71) and correlation between the methods (r = 0.969) and the absence of statistically significant differences in the t-test (Fig. 4A). However, despite the minimal systematic bias (bias = 0.01) in the Bland-Altman analysis (Fig. 4B), the limits of agreement were ±0.7, which corresponds to a relative error, so although the methods demonstrate statistical consistency, their compliance cannot be considered satisfactory for practical application.

As a result of assessing the body type, it was revealed that on average, according to the anthropometric method, the body type of females is represented by the endomesomorphic type (4.6 – 4.1 – 2.3), and according to the BIA method - meso-endomorphic (4.2 – 4.9 – 2.3).

Analysis of the Bland-Altman graph of endomotrphy revealed a slight systematic bias (bias = −0.39), indicating a slight underestimation of the values of one method relative to the other. The limits of agreement range from –3.33 to +2.55, demonstrating a significant variation in differences between the methods (Fig 6A). A weak correlation (r = 0.227) and a low concordance coefficient (pc = 0.21) indicate the absence of a pronounced proportional error (Fig 6B). However, the wide agreement limits and the negative bias indicate that there are significant differences between the methods that do not depend on the magnitude of the measured parameter. The results obtained emphasize the need for careful interpretation of the data and indicate the limited interchangeability of the methods in clinical practice.

**Fig. 5:**
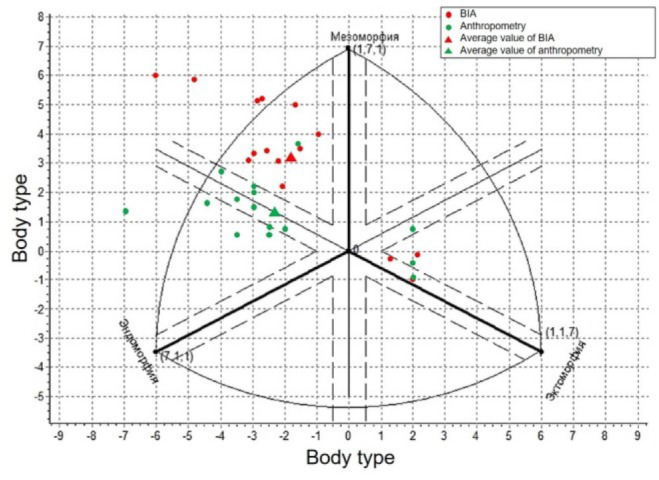
Comparison of female somatotyping results by the Heath-Carter method using BIA and anthropometry. BIA – bioelectrical impedance analysis.

**Fig. 6:**
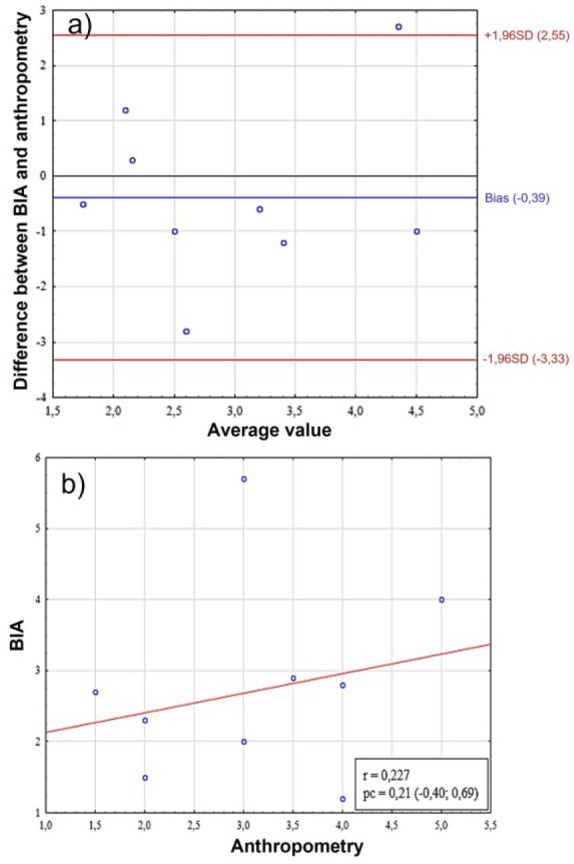
Comparative analysis of endomorphy scores in young men. A – assessment of bias using the Bland-Altman method, the Y-axis shows the “BIA-Anthro” difference in endomorphy scores, B – scatter plot between two methods for assessing somatotype. BIA – bioelectrical impedance analysis, SD (standard deviation), r – Pearson correlation coefficient, pc – Lin correlation concordance coefficient.

**Fig. 7:**
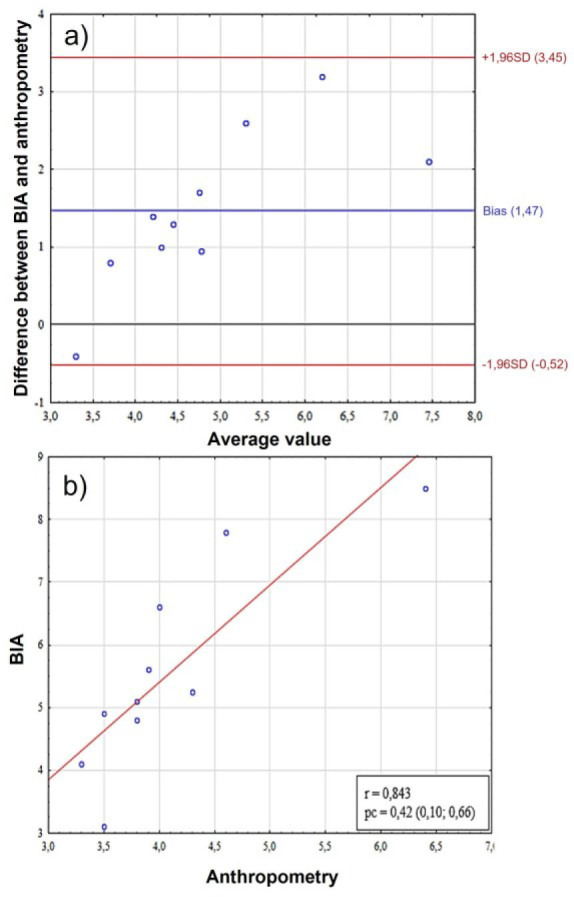
Comparative analysis of mesomorphy scores in male. A – assessment of bias using the Bland-Altman method, the Y-axis shows the “BIA-Anthro” difference in mesomorphy scores, B – scatter plot between two methods for assessing somatotype. BIA – bioelectrical impedance analysis, SD (standard deviation), r – Pearson correlation coefficient, pc – Lin correlation concordance coefficient.

On the other hand, the assessment of mesomorphy scores also showed a lack of concordance – the bias according to the Bland-Altman method was 1.47 (95%CI = –0.52, 3.45), r = 0.834, pc = 0.42 (95% CI = 0.10; 0.66) (Fig. 7).

**Fig. 8:**
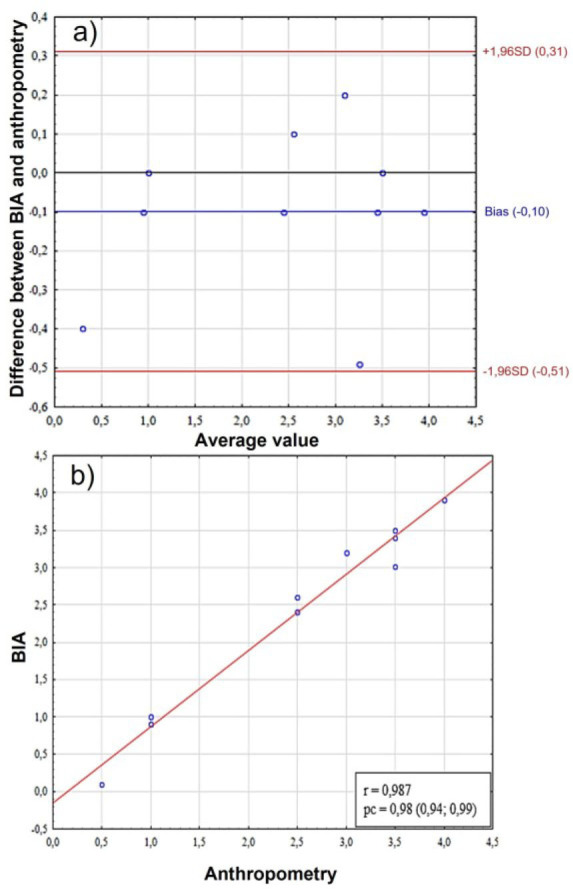
Comparative analysis of ectomorphy scores in male. A – assessment of bias using the Bland-Altman method, the Y-axis shows the “BIA-Anthro” difference in ectomorphy scores, B – scatter plot between two methods for assessing somatotype. BIA – bioelectrical impedance analysis, SD (standard deviation), r – Pearson correlation coefficient, pc – Lin correlation concordance coefficient.

Ectomorphy measurement scores, as in the case of females, were also rated as having the greatest consistency, pc = 0.98 (95%CI = 0.94, 0.99), despite greater mean bias (–0.10, 95% CI = –0.51; 0.31) (Fig. 8).

The presented data demonstrate a purely mathematical consistency of the methods, which determines their minimum discrepancy by default. The Bland-Altman graph shows almost complete agreement between the results: the systematic bias is negligible (bias = –0.10), and the limits of agreement are exceptionally narrow (–0.51 to +0.31).

The somatotype assessment showed that both methods, BI and anthropometry, demonstrate similar, but not absolutely identical results in the classification of participants' somatotypes. The greatest discrepancies are observed in the assessment of endomorphic and mesomorphic characteristics, while the ectomorphic component is usually determined by both methods more consistently.

While both methods revealed a comparable balance between endomorphy and mesomorphy, the anthropometric approach (3.1 – 3.8 – 2.4 ) showed a slightly greater emphasis on characteristics related to adipose tissue due to measurements of skin folds, whereas BIA (2.1 – 5.6 – 2.4) demonstrated a higher sensitivity to muscle development through analysis electrical impedance. It is noteworthy that these methods gave the same ectomorphic estimates (2,4), which indicates a consistent assessment of linear body proportions.

**Fig. 9:**
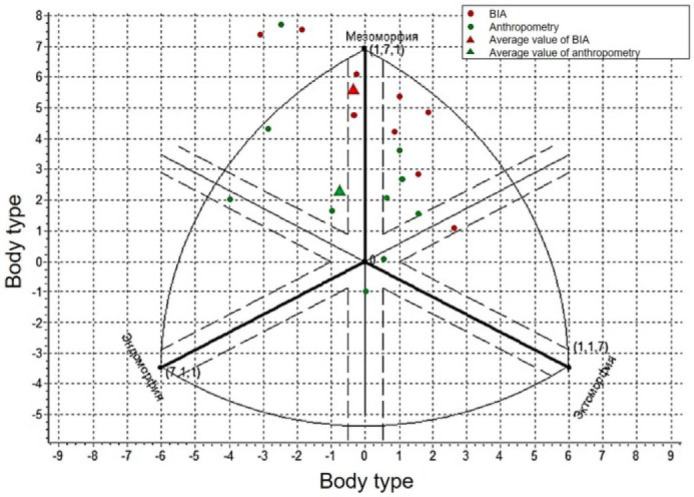
Comparison of somatotyping results of male by Heath-Carter method using BIA and anthropometry.

These systematic but predictable differences reflect the measurement orientation inherent in each method – anthropometry records the external dimensions of the body and subcutaneous fat, while BIA evaluates body composition using tissue conduction (Fig. 9).

## Discussion

The use of BIA as a tool for somatotyping is a relatively new approach in morphology. The key difference of this approach is the calculation using predictive equations based on the measured active resistance of the body [12, 13]. This method has several disadvantages, such as strict requirements for standardization of measurement conditions. On the other hand, this method significantly reduces the time spent on analysis. The anthropometry method is less dependent on measurement conditions but requires certain skills. The measurement must be carried out by the same person, using the same anthropometric instruments.

The task is also complicated by the large number of recommendations based on which measurements are taken. Thus, there is an approach to taking measurements described by Eston and Reilly [14,15,16,17], and the ISAK method will be different from other guidelines. This fact must be taken into account by researchers when concordating data in somatotyping. BIA may become the most unified solution for data interpretation.

There are well-known works comparing the somatotype of men and women [18-19]. Thus, when calculating ectomorphy scores, there are no differences between the two methods in both sexes, but the assessment of mesomorphy scores has significant differences - in both males and females, mesomorphy scores were higher when using BIA, and in males, mesomorphy scores obtained using BIA were 1.5 times higher than for females.

This phenomenon was especially noticeable when analyzing body type on the Heath-Carter graph (Fig. 9), where one can observe a serious disparity between the BIA and anthropometry data. Most of the data obtained during anthropometric measurements ranges from –2 to 2 along the X–axis and from 0 to 3 along the Y–axis, i.e. they are located closer to the center of the graph, while the data obtained during BIA are located closer to the periphery.

Despite the differences, both methods demonstrate consistency in assessing body type in male when using both methods, body type was assessed as meso-endomorphic (according to average values), however, BIA shows a higher level of mesomorphy scores in male.

The data obtained during the assessment of the somatotype of females also show similar results for assessing the body type, however, a shift towards mesomorphy is also observed when using BIA.

## Conclusion

Somatotyping is a very important component of medical, biological and sports sciences. However, methods for assessing somatotypes may differ from each other. Thus, the present study showed that the data obtained from somatotype applying anthropometry and bioelectrical impedance analysis have differences and a low level of consistency, but body type is estimated in approximately equal ranges.

BIA, to a greater extent, shows an increased level of mesomorphy scores in both males and females, and the difference in males is more pronounced. On the other hand, endomorphy scores are lower when assessing the somatotype using the anthropometric method. Ectomorphy scores are scored equally by both methods, demonstrating good agreement.
